# Massive open online course for Brazilian healthcare providers working with substance use disorders: curriculum design

**DOI:** 10.1186/s12909-020-02162-w

**Published:** 2020-07-29

**Authors:** Erika Pizziolo Monteiro, Henrique Pinto Gomide, Eduardo Remor

**Affiliations:** 1 Institute of Psychology, Universidade Federal do Rio Grande do Sul (UFRGS), Rua Ramiro Barcelos, 2600, Porto Alegre, Rio Grande do Sul 90035-003 Brazil; 2 Department of Education, Universidade Federal de Viçosa (UFV), Av. Purdue s/n, Viçosa, 36570-000 Minas Gerais Brazil

**Keywords:** Substance use disorders, Health personnel attitudes, Empathy, Communication skills, Personnel training

## Abstract

**Background:**

Interpersonal and technical skills are required for the care of people living with substance use disorders. Considering the applicability and usability of online courses as continuing professional education initiatives, this study aimed to describe the content design process of an introductory-level healthcare-centered Massive Open Online Course (MOOC).

**Methods:**

The content of the course was informed through needs assessment, by using three sources: (a) narrative literature review, (b) Delphi health experts panel consensus, and (c) focus groups conducted with people living with substance use disorders. The data from the empirical research phases were analyzed through qualitative Thematic Analysis.

**Results:**

The product of this research project is the introductory-level Massive Open Online Course “Healthcare: Developing Relational Skills for the Assistance of People Living with Substance Use Disorders” which approaches health communication and empathetic relational professional skills as a means of reducing stigmatization of people living with substance use disorders.

**Conclusions:**

Diverse strategies for designing distance education initiatives have to consider different views on the subject being approached in such courses. The product presented in this paper has the potential to be an educational tool for topics traditionally not addressed in Brazilian continuing education and can be used as a model to the design of online courses directed to the development of work-related skills for the healthcare professions.

## Background

Substance use disorders are a major public health concern affecting millions of people worldwide [1]. This condition requires evidence-based treatment delivered by a multi-professional team and has distinct characteristics [2], like relapses, which makes it more challenging to manage [3].

Interpersonal and technical skills are required for the care of people living with substance use disorders. Patient-Centered Care is recommended as a model for planning and conducting health care [4]. This is built on principles like viewing the patient as a singular person and perceiving the health professional-patient relationship as a partnership grounded on care and trust, considering patient characteristics as well as professionals’ competencies and attitudes [5, 6].

The Competence Benchmarks for Professional Psychology [7, 8] indicates relational aspects as fundamental competencies for working with patients with substance use disorders. To build rapport, the literature suggests that relational abilities like empathy and communication skills are essential for promoting behavior change [9, 10].

Since practitioners from different health care settings work with people with substance use disorders, it is important to understand the role of training programs in approaching the development of professional competencies needed for providing mental health services [11]. Training is critical to the development of a diverse and well-trained workforce with the skills needed to care for people living with substance use disorders [12]. Therefore, contextually and empirically-based training programs designed to develop such skills are required [13, 14].

Courses targeting work-related skills have the potential for higher enrollment and completion rate [15, 16]. Skill training can be defined as a proposal for the development of a series of discrete elements that build complex behavior and that leads or helps build a relationship between the health professional and patients by addressing when and how to use certain communication strategies [17]. Considering this definition, it becomes necessary to define training strategies, using different applied approaches that allow the development of target skills [18, 19].

Needs assessment is essential for planning continuing educational programs and can contribute to practical application of content to professional activities [20]. Educational needs are generalized to target audience considering standards of practice [20] and should be drawn from diverse perspectives [21]. The approach chosen for the purpose of the present study was the development of an introductory-level Massive Open Online Course (MOOC) as an accessible tool for health professionals and social workers with little or no previous experience in the course content.

The course content was developed using evidence-based information on stigmatization processes related to substance use disorders and its negative effects and the role of health communication strategies and empathy in the assistance of people living with substance use disorders. The content was adapted for the structure of the healthcare system in Brazil, considering the low availability of continuing education courses targeting variables as communication skills and empathy for the development of therapeutic relationships with people living with substance use disorders.

This study describes the content design process of a MOOC by combining current scientific literature and inputs from experts on the alcohol and other drugs and people living with substance use disorders undergoing treatment in a community-based facility.

## Methods

### Research design

The course design was based on guidelines for intervention development proposed by Kok and collaborators [14] and participatory research [22] as a needs assessment approach.

The ADDIE instructional design approach (i.e., Analysis, Design, Development, Implementation, and Evaluation) [23] was also used as a guideline for course design and multimedia content creation. The ADDIE process is commonly used in the planning of instructional courses having a student-centered approach. The phases are: (a) Analysis, a needs assessment phase, where a field or topics worth pursuing as a training program are identified, (b) Design, a research phase in which the strategies that will be employed to achieve the learning objectives are defined, (c) Development, instructional course content construction characterized as drafting, production and evaluation, (d) Implementation, course dissemination to the target audience and (e) Evaluation, operationalized during the implementation phase as a process evaluation. This paper focuses on curriculum development applying the Analysis, Design and Development phases described above.

Considering the need for the feasibility of the content produced to the care context of people living with substance use disorders, consultation from various sources is essential to include different views of what accomplishes for quality care [22]. We divided the creation of the course syllabus into three studies (phases) carried out separately and described in Table 1.

**Table 1:** Studies conducted for designing the MOOC syllabus

Studies	Aims
**Study 1:** a narrative review [5] on topics related to the course	To summarize the scientific evidence and contextual content used for theoretical and practical decisions related to the course
**Study 2:** a Delphi group of experts in substance use disorders [24, 25]	To survey experts input on practical content to be included in the course
**Study 3:** a focus group [26]	To elicit the views and experiences of people under treatment for substance use disorders in order to include these views in the course discussions

To conduct the studies, we used as reference the guidelines for qualitative research published by the American Psychological Association (APA, [27] and accessed the directions provided by Salmon [28].

The content of the MOOC was organized in courseware. Four modules were developed, namely: (1) Drug Policies and Substance-related Care in the Brazilian Context, (2) Attitudes related to People living with Substance Use Disorders, (3) Health Communication Skills for Professionals in the Substance Use Field and (4) Empathy as a Therapeutic Tool. Visuals were added to the materials which were proofread for spelling and reference.

### Study 1

A narrative review about the stigmatization processes, healthcare providers communication and empathy-related skills for the assistance of people living with substance use disorders.

### Study design

The literature review was conducted in order to summarize the scientific evidence and contextual content used for theoretical and practical decisions related to the course.

We performed a narrative review on epidemiology, policies and the Brazilian healthcare system for substance use disorders, the stigmatization process of substance use as a health condition and the professional skills needed to work with people living with substance use disorders [5]. This study was conducted as an Analysis and Design phases from the ADDIE instructional design approach [23].

### Procedures

Searches for academic literature published in English or Portuguese were performed in the PubMed, PsychINFO and Virtual Health Library Brazil databases, as well as on official pages of the Brazilian National Drug Policy Secretariat, Brazilian National Health Department and the Brazilian Federal Council of Psychology.

Medical Subjects Headings (MeSH), Thesaurus from APA and Health Sciences Descriptors (DeCS, Brazilian acronym) were consulted to refine the search terms used in the review. The search words are described in Table 2.

**Table 2:** Search words for conducting the narrative review

Databases	Search words
Medical Subjects Headings (MeSH)	(1) substance related disorders and (2) attitude of health personnel or (3) health knowledge, attitudes and practice or (4) social stigma or (5) empathy or (6) health communication
Thesaurus (APA)	(1) substance use disorder and (2) health personnel attitudes or (3) health knowledge or (4) stigma or (5) empathy or (6) communication skills training
Health Sciences Descriptors (DeCS, Brazilian acronym)	(1) *transtornos relacionados ao uso de substâncias* and (2) *conhecimento, atitudes e prática em saúde* or (3) *estigma social* or (4) *empatia* or (5) *comunicação em saúde*

In conducting the literature research, no date limits were established, but considering the relevance of potential references, recent publications were favored in an attempt to use relevant current information for course content development.

Research articles and theoretical material were included, which were: a) applicable information for clinical work regarding people living with substance use disorders, b) published by peer-reviewed journals, organizational publishers or that included theoretical frameworks systematically used in the substance use disorders field and c) written in Portuguese or English.

The review was extended by consulting the reference section of documents. Since the theoretical approach used in the course was defined as Patient-Centered Care, theoretical and empirical articles specific to this theme were searched and evaluated.

### Study 2

Delphi expert consensus about treating people living with substance use disorders.

### Study design

This study strategy was characterized as a mixed-method Delphi study to survey experts input on practical content to be included in the course. The Delphi method is a way of eliciting and refining information from a group [24, 25].

First, qualitative data analysis was performed on experts’ opinions and ideas collected by open-ended interviews. Then, the same experts evaluated the analyzed data by answering questions on a Likert scale.

### Participants

Twenty healthcare professionals with experience in substance use disorders were invited by e-mail. E-mails were obtained from the literature analyzed in study 1. Invitations were made to achieve diversity among participants in terms of profession and location. Eight responses were collected in the first round and six in the second.

### Procedures: Round 1

An online survey was made available in SurveyMonkey® (SurveyMonkey Inc., San Mateo, California, USA, https://surveymonkey.com/), containing an electronic informed consent form, open-ended questions, and a sociodemographic questionnaire. The questions were: (1) What professional skills do you think are necessary for the assistance of people living with substance use disorders? (2) There are various beliefs and opinions about substance use disorders. Given your experience, what beliefs do professionals you work with have about people living with this condition? (3) In your view, what would be the best way to approach such beliefs? (4) When you look at your colleagues working on the substance use disorders field, which attitudes do they exhibit? (5) In your opinion, which professional attitudes involved in caring for people living with substance use disorders should be displayed? (6) What is the best way to communicate with people living with substance use disorders?

### Data analysis: Round 1

Once the professionals’ answers were received, they were qualitatively analyzed through the Thematic Analysis procedures by author EPM and a research assistant. For the purposes of this study, rich thematic description strategies were performed, through which a broader variety of themes is extracted from the data [29]. The NVivo® 12 software (QSR International Pty Ltd., Victoria, Australia) was used to assist in the data analysis.

### Procedures: Round 2

Closed-questions were produced for the themes and subthemes generated by the qualitative data analysis.

Participants were asked to evaluate the proposed subthemes in terms of relevance and suitability for inclusion in online training for health and social care professionals with an interest in the substance use disorders field. The response options, regarding the suitability criterion, were: very inadequate, inadequate, neither inadequate nor adequate, adequate and very adequate. Regarding the relevance criterion, the following options were available: very irrelevant, irrelevant, neither irrelevant nor relevant, relevant and very relevant.

This procedure was performed to reach consensus among the consulted professionals [24, 25] and also used as a means to validate the qualitative analysis process performed in round one. In other words, the second round of procedures for the Delphi method facilitates consensus and is classified as a means of rigor and trustworthiness for the qualitative analytic process, since the themes extracted from the data collected in the first round are evaluated by the same participants who answered the open-ended questions in the first place.

### Data analysis: Round 2

We performed a descriptive data analysis on participants’ agreement on the relevance and suitability of the extracted themes. For reporting purposes, the mean concordance for each theme was calculated based on the information given for the subthemes composing a theme.

There were five response options to each criterion yet, for presentation purposes, they were described as binary. Regarding the suitability criteria, very inadequate, inadequate and neither inadequate or adequate were considered as inadequate and adequate and very adequate were considered as adequate. Related to relevance, very irrelevant, irrelevant and neither irrelevant or relevant were considered as irrelevant and relevant and very relevant were considered as relevant. The subthemes with an agreed percentage of 60% or more on both criteria were included in the course syllabus.

### Study 3

Focus Group: Service users’ perceptions about their treatment.

### Study design

A focus group was designed to identify the service users’ perceptions of how they were welcomed and treated by the various professionals that they have met during their treatment history to include these views in the course discussions. Research procedures regarding this study followed the recommendations from Krueger and Casey [26]. This study is the last of three research strategies for Analysis and Design as proposed in a course instructional framework [23].

### Participants

Patients undergoing treatment in a Psychosocial Care Center for people living with substance use disorders (CAPS AD, Brazilian acronym, government public service), in a metropolitan region of south of Brazil, were invited to participate in the research activity by researchers in a face-to-face assembly held as part of the routine work of the data site collection facility.

Seven participants agreed with the research invitation and composed the focus group. The method used was a convenience sampling process and the criteria for selection were the interest in sharing information on how the relationship between themselves and health professionals and social workers was formed in their treatment history. The sample was evaluated as sufficient considering the focus group research procedures described by Krueger and Casey [26].

### Procedures

For their personal experience with regard to the substance use disorder treatment process, people in treatment in psychosocial care network services are considered critical informants who can, therefore, contribute to the understanding of some important aspects related to the healthcare of people living with substance use disorders.

The research objectives and procedures were presented to coordinators of the Psychosocial Care Center (CAPS AD) after a meeting with the multi-professional team. After consent, the focus group was conducted by the author - EPM - and a research assistant. The research assistant was responsible for the generation of the field diary, based on the observations made during the focus group. The discussion was recorded and later transcribed for qualitative data analysis purposes.

A semi-structured script was used and the following steps were followed: (1) presentation of the researchers and research objectives, (2) presentation of the informed consent form, requesting the signature of those who agreed to participate in the study, (3) self-presentation of the participants, including their treatment history, (5) questions that aimed to investigate the relationship established between participants and the various health and social care professionals with whom they have interacted during their treatments for substance use disorders, (6) acknowledgment and group closure.

### Data analysis

Thematic Analysis [29] on the focus group data was conducted using NVivo Pro 12 software (QSR International Pty Ltd., Victoria, Australia). As a strategy regarding analytic rigor and trustworthiness, the themes extracted from the focus group data were evaluated from a triangulation point of view, being added as a result from this study the ones who were consistent with patient-centered care approach dimensions [6] and in agreement with the themes extracted from the Delphi panel expert consensus.

## Results

### Study 1

Considering the research articles, the official manuals published in Brazil concerning the care of people living with substance use disorders and materials such as books and theses, four modules were developed: (1) Drug Policies and Substance-related Care in the Brazilian Context, (2) Attitudes related to People living with Substance Use Disorders, (3) Health Communication Skills for Professionals in the Substance Use Field and (4) Empathy as a Therapeutic Tool. Table 3 contains modules content, number of articles included as references in the MOOC and their range of publication dates.

**Table 3:** Modules content

Modules	Content	Number of included articles	Years of Publication
Drug Policies and Substance related Care in the Brazilian Context	Brazilian National Drug Policy Treatment strategies published by the national government National epidemiology data The Brazilian health and social care systems related to the alcohol and other drugs field International action and assistance plan for helping people living with substance use The United Nations 2030 Agenda The available services including prevention and treatment	8	From 2007 to 2020
Attitudes related to People living with Substance related Disorders	Theoretical perspectives on health provider attitudes related to people living with substance use disorders Stigmatization processes of people living with substance use disorders Attribution theory and its relations with the alcohol and other drugs field Brickman’s Model and the individual responsibility for healthcare Substance use disorders and media coverage Consequences from the stigmatization process Challenges associated with working in the alcohol and other drugs field	16	From 1980 to 2018
Health Communication Skills for Professionals in the Substance Use Field	Therapeutic relationship between patient and health care provider Therapeutic communication Communication problems Interpersonal skills Motivational interview	20	From 1983 to 2018
Empathy as a Therapeutic Tool	Empathy definition Benefits related to empathic health care Contextualized assistance Patient centered care Health provider selfcare	10	From 2003 to 2020

### Study 2

Participant description.

The experts recruited for the Delphi panel sociodemographic characterization is described on Table 4.

**Table 4:** Study strategy 2 participant description

Sociodemographic information	Total of 8 participants
Age	Mean of 40,6 years
Gender	Men (*N* = 4)
	Women (*N* = 4)
Profession category	Psychology (*N* = 6)
	Medicine (*N* = 1)
	Occupational Therapy (*N* = 1)
Degree	Postgraduate (*N* = 8)
Place of Work	Universities (*N* = 5) Psychosocial Care Center for patients living with substance use disorders [(CAPS AD, Brazilian acronym, government public service), (*N* = 1)] Social Assistance Centers [(CRAS, Brazilian acronym, government public service), (*N* = 2)]
Time working on alcohol and other drugs	Mean of 14 years (4 years minimum and 28 maximum)
Location on Brazil	São Paulo (*N* = 3)
	Minas Gerais (*N* = 3)
	Maranhão (*N* = 1)
	Rondônia (*N* = 1)

### Rounds 1 and 2

Themes and subthemes extracted through Thematic Analysis from information provided by the Delphi method panel experts on round 1 of procedures and evaluated considering the suitability and relevance for inclusion in course content constitute the results from study 2 strategy. These results are presented in Table 5.

**Table 5:** Themes and sub-themes from Delphi panel and frequency of agreement for inclusion in course syllabus

Theme	Subthemes	References	Adequate	Relevant
Negative Beliefs	Stigmatizing perception	6	83,33%	100%
	Patient’s personal characteristics as determinants of treatment	3	100%	66,67%
	Abstinence as the only option	1	100%	83,33%
	Substance use as a cause of vulnerabilities	1	100%	83,33%
	Strictly medical view of the disorder	1	66,67%	66,67%
Positive Attitudes	Empathetic reception	6	100%	100%
	Humanization of treatment	2	83,33%	83,33%
	Rapport building	2	83,33%	83,33%
	Treatment customization	3	83,33%	83,33%
	Respect	2	100%	100%
	Co-responsibility for treatment	1	83,33%	83,33%
	Harm reduction as a possibility of action	2	83,33%	83,33%
Professional Competencies Needed	Empathy	9	100%	100%
	Social context assessment to propose action	2	83,33%	83,33%
	Technical knowledge	6	83,33%	83,33%
	Communication skills	3	100%	100%
Communication Skills	Intently listening	4	100%	100%
	Clarity in information sharing	1	100%	100%
	Providing feedback	1	100%	100%
	Assertiveness	2	100%	100%
	Patient comprehension assessment	1	100%	100%

### Study 3

The results that emerged from the focus group with people living with substance use disorders indicate and highlight the core role that community health services and the relationship established between health professionals and service users to better health outcomes. Themes extracted from this research method are described in Table 6 and were used to tailor the theoretical and practical contents included in the MOOC.

**Table 6:** Themes extracted from the Focus Group

Themes	Description
Treatment customization	Consider the biopsychosocial aspects related to each patient to propose contextualized actions
Importance of health services for treatment	The existence of health services in the public care network as a support for symptom reduction or search for abstinence
Importance of Occupational Therapy	Occupational activities as a therapeutic tool enabling patient development of useful skills in contexts beyond the health service
Reception offered by service professionals	Respect offered by professionals every day assisting in the process of adherence to the unique therapeutic plan
Clear rules for service routine	Establishment of treatment service routines
Knowledge about addiction as a professional aspect	Technical professional knowledge about substance use disorders as an essential skill
Attentive listening	Professional participation in the treatment process proving to be involved in the work process
Healthcare as a safe space	Healthcare service as a safe place where risk factors for substance use are minimal
Build rapport with professionals	Collaborative relationship between health professional and patient
Emotional support as a necessary aspect of treatment	Professional competency for approaching patient emotional distress

### Curriculum as a main result

The main product of this research paper is the introductory-level MOOC “Healthcare: Developing Relational Skills for the Assistance of People Living with Substance Use Disorders”, built as a continuing education initiative. The MOOC is composed by videos, modules and assignments and Table 7 contains the course syllabus.

**Table 7:** MOOC syllabus

	Module 1	Module 2	Module 3	Module 4
Themes	Drug Policies and Substance related Care in the Brazilian Context	Attitudes related to People living with Substance related Disorders	Health Communication Skills for Professionals in the Substance Use Field	Empathy as a Therapeutic Tool
Videos	Video 1: Module Presentation	Video 3: Module Presentation	Video 5: Module Presentation	Video 7: Module Presentation
	Video 2: Health Promotion in the Field of Alcohol and other Drugs	Video 4: Negative and Positive Attitudes towards People living with Substance Related Disorders	Video 6: Health Communication Models	Video 8: Interview with a Phycologist and Researcher on the Empathy Topic
Readings	Reading 1	Reading 2	Reading 3	Reading 4
Assignments	Assignment 1: Discussion Forum (The Care Network for People living with Substance Related Disorders) Assignment 2: Multiple Choice Quiz	Assignment 3: Discussion Forum (The Challenges of the Alcohol and other Drugs Health Care) Assignment 4: Discussion Forum (Models of Substance use Perception) Assignment 5 (Tool 1): Positive Stories Assignment 6: Multiple Choice Quiz	Assignment 7: Discussion Forum (Communication Strategies) Assignment 8: an interview practice transcription Assignment 9: Multiple Choice Quiz	Assignment 10 (Tool 2): Substance Related Disorders Caring: A Guide for Health Professionals and Social Workers Assignment 11: Discussion Forum (Feedback on the guide provided in the module) Assignment 12: Multiple Choice Quiz

Thereadings contain the same title as the modules

## Discussion

The overall aim of the MOOC is to improve healthcare quality through the use of evidence-based care strategies [30] that are also endorsed by specialists acting in the Brazilian alcohol and other drugs field. Content of an introductory-level MOOC was identified by using three strategies: (a) narrative review, (b) Delphi consensus, and (c) focus groups. These strategies were used as complementary approaches, tapping the diverse perspectives of researchers, health professionals and people living with substance use disorders undergoing treatment. The results obtained from them are consistent with each other, enabling the development of scientifically sound and contextually applicable content. There is a need for evidence-based recommendations to meet the practical challenges of everyday healthcare practice and online continuing education initiatives have to be built on relevant current information [31]. The literature on the development of continuing education initiatives emphasizes that online courses should be practical, assisting in enhancing work-related skills [32].

The literature on the topics included in the MOOC described in the present paper - stigma, health communication skills, and empathy - corroborates the themes and subthemes extracted from the qualitative research strategies implemented through Delphi consensus and focus group.

From the Delphi consensus regarding the stigmatization process associated with substance use disorders, two themes can be emphasized: negative beliefs and positive attitudes. Stigma is characterized as labeling, stereotyping, and separation of others from oneself, leading to status loss and discrimination [33, 34]. Subthemes extracted from the Delphi consensus were related to attitudes such as the view that the patient’s personal characteristics are determinant of treatment, that abstinence is the only option, that substance use disorders are the cause of vulnerabilities and a strictly medical view of the disorder. These are examples of the labeling and stereotyping as components of the health condition-related stigmatization process [35]. These are negative views not only from the general public but also from healthcare providers, which have potentially harmful consequences for the assistance of people living with substance use disorders, undermining access to diagnosis, treatment and prevention practices and better health-related outcomes [36].

Stigma reduction strategies were associated with positive attitudes cited by the consulted experts. These strategies were also linked to the themes extracted from the focus groups. In a review of stigma reduction strategies conducted by Nyblade and coauthors [35], a large number of interventions (37 out of 42) targeted topics were identified like the knowledge of health condition, knowledge of stigma and ability to manage the health condition. Professional competencies and communication skills extracted from our data were related to treatment customization, importance of Occupational Therapy, reception offered by service professionals, the clarity of service rules and attentive listening. These competencies are necessary to build rapport and to demonstrate emotional support required for working in the substance use disorders field.

Synnot and coauthors [37] aimed at defining the priorities for the conduction of Cochrane Systematic Reviews in which targeted audience composed of stakeholders discussed current needed research initiatives. The results indicate that between the five topics extracted from the qualitative data, there is promoting patient-centered care, the theoretical approach underling the online educational initiative described in this paper. In the same research, 12 priority problems were the jumpstart point of the discussion which included the need for better understanding from healthcare providers of patient-centered concept and practices, a better way of sharing information with patients, the involvement of service users in the decision-making process, participating actively according to their priorities. We emphasize that all of these topics were covered in the MOOC presented in this paper.

The results presented here are related to the curriculum development process in similarity with previous papers published in the literature (for example, [38]). A previous study comparing online and in-person educational strategies concludes that both initiatives were effective in generating readiness to practice in the field of alcohol and other drugs [39]. Thus, the hypothesis to be tested in a future study considers the probable effectiveness of the course [40] in reducing stigmatizing attitudes towards people living with substance use disorders through the development of greater professional communication skills and empathetic posture for planning and conducting care as a patient-centered approach.

At the time of this manuscript’s submission, the MOOC was at a production phase by a university distance education team. Once completed the course will be disseminated among public and private healthcare and social assistance Brazilian facilities.

MOOCs have the potential to reach a broad audience, as they are self-paced and can be accessed at any time. These characteristics might improve the retention of students - especially health professionals who often work long hours. There are other educational initiatives presented as massive open online strategies on the health field operationalized in Latin America [41].

The educational tools influence the learning process and need to be adapted to the context of the target course audience. In that matter, various sources of information can contribute to the development of applicable content with evidence of effectiveness. The use of booklets, videos, and interactive tasks seeks to engage the audience, providing spaces for connected learning. Considering that acting in the field of alcohol and other drugs should be done by multi-professional teams, these activities have the potential to enable the development of integrated work skills [32]. The learning exercises, such as discussion forums, were based on the connectivism learning theory - a pedagogical approach in which interactions between learners contribute to knowledge acquisition [42].

### Limitations

Regarding the study of strategy 1, a narrative review without a systematic evaluation of the research literature was conducted. This type of review can be influenced by subjective views from the researchers when selecting the references for inclusion in the MOOC content. For the study of strategy 2, a Delphi consensus method, there was a significant sample loss between recruitment and inclusion, 20 participants were recruited and 8 agreed to participate composing a convenience non-representative sample. As far as sociodemographic information related to the sample, no social works were recruited, so participation was restricted to psychologists, doctors and occupational therapists, some of them working in social assistance facilities. The data collected from the study of strategy 3, the focus group, was analyzed by one author and a research assistant, so there was no comparison basis for the results extracted from the qualitative corpus of information. Still an attempt to reduce biases was made by using three research strategies from diverse information sources.

## Conclusions

Diverse strategies for designing distance education initiatives have to consider different views on the subject being approached in such courses. In the present paper, topics such as healthcare providers stigmatized views, communications skills and empathy in the field of alcohol and other drugs were researched through complementary perceptions enabling the construction of scientifically-based and practically applicable educational multimedia content which will be evaluated in terms of its efficacy in future research.

The product presented in the present paper, the MOOC “Healthcare: Developing Relational Skills for the Assistance of People Living with Substance Use Disorders” has the potential to be an educational tool in topics traditionally not addressed in continued education strategies in the Brazilian healthcare process and can be used as a model to the design of online courses directed to the development of work-related skills for the healthcare professions.

## Data Availability

The datasets generated and/or analysed during the current study are not publicly available due to the personal and/or qualitative nature of the information provided by the research participants, but are available from the corresponding author on reasonable request.

## Abbreviations

MOOC: Massive Open Online Course
ADDIE: Analysis, Design, Development, Implementation, and Evaluation
APA: American Psychological Association
MeSH: Medical Subject Headings
DeCS: *Descritores em Ciências da Saúde* (Health Science Descriptors)
CAPSAD: *Centro de Atenção Psicossocial Álcool e Drogas* (Psychosocial Alcohol and Drugs Care Center)
CRAS: *Centro de Referência de Assistência Social* (Social Assistance Reference Center)
